# A New Design of Recycled Ethylene Propylene Diene Monomer Rubber Modified Epoxy Based Composites Reinforced with Alumina Fiber: Fracture Behavior and Damage Analyses

**DOI:** 10.3390/ma12172729

**Published:** 2019-08-26

**Authors:** Alaeddin Burak Irez, Georges Zambelis, Emin Bayraktar

**Affiliations:** 1 LMT, ENS-Cachan, CNRS, Université Paris-Saclay, 94235 Cachan, France; 2 Airbus Helicopters, 1 Place du Générale Valérie André, 93440 Dugny, France; 3 SUPMECA-Paris, School of Mechanical and Manufacturing Engineering, 93400 Saint Ouen, France

**Keywords:** three-point bending, fracture toughness, toughening mechanisms, alumina fiber, recycled rubber, SEM

## Abstract

This study proposes a new design of lightweight and cost-efficient composite materials for the automotive industry using recycled fresh scrap rubbers (EPDM (ethylene propylene diene monomer) rubbers), epoxy resin and alumina (Al_2_O_3_) fibers (AF). Three-point bending tests were conducted to investigate fundamental mechanical characteristics and then experimentally obtained moduli were compared with a modified Halpin–Tsai model. In addition, tests were carried out to study the fracture characteristics of the composites. Then, a practical numerical study was carried out to observe the evolution of the strain energy release rate along the crack front. Mechanical test results showed that the reinforcement with AF improved the fracture toughness of these novel composites for low rubber contents. Besides, increasing recycled EPDM rubber content degraded the mechanical resistance and strain at break of the composites. Moreover, numerical studies indicated that energy release rate showed some variations along the specimen thickness. Toughening mechanisms were evaluated by scanning electron microscope (SEM) fractography. Typical toughening mechanisms observed were fiber bridging and shear yielding. By considering the advantageous effects of AF on the novel composites and cost efficiency under favor of recycled rubbers, these composites are promising candidates to manufacture the various components in automotive industry.

## 1. Introduction

Material recycling currently attracts considerable worldwide attention because of environmental and economic issues. Among the recyclable materials, rubber has a prominent place in many sectors. After completing its service life, a deposit of scrap rubber creates environmental issues including soil contamination and mosquito habitat [1,2,3]. The environmental impact of scrap rubber can be reduced if recycled, and this recycling can be achieved by using scrap rubber in novel composite manufacturing. Recycled rubber containing composites can offer considerable benefits over current composites. Recycled rubbers can improve the toughness and impact resistance as well as they offer a major cost advantage for the manufactured composites. In this regard, proposing a low cost and lightweight material for the automotive industry constitutes the main objective of this study. 

As the matrix of these polymer-based composites, epoxy is considered an appropriate thermosetting polymer due to its ease of processing, high stiffness, large specific strength, environmental stability and relatively low cost [4]. However, a highly cross-linked network of epoxy comes with some undesirable properties such as brittleness. Therefore, incorporation of hard particles such as alumina fibers is done to toughen the epoxy matrix. The addition of AF promises the desired mechanical characteristics in case of a homogeneous distribution due to beneficial structural characteristics, such as fiber interlocking effect. Also, AF are environmentally-friendly and low-cost. Last but not least, AF have high compressive strength and also the elastic modulus of AF in radial direction is higher than carbon fiber that offers these composites a further benefit. Accordingly, cost efficient hybrid composites can be manufactured based on epoxy with the incorporation of recycled rubber and AF [5,6].

Although the expectations mentioned above are fulfilled, recycled rubber does have certain disadvantages. First, vulcanization is conducted during rubber manufacturing by the rubber suppliers to enhance the rubber characteristics and during vulcanization, crosslinks are generated in between the free links of rubber and externally diffused sulphur atoms. However, when recycled rubber that is already vulcanized is used in composite manufacturing, it is hard to establish chemical bonds between epoxy matrix and recycled rubber because of the absence of rubbers’ free links. Consequently, the absence of chemical bonding causes certain interface problems. Moreover, because of the high van der Waals forces between recycled rubber particles, agglomerations can be observed in the microstructure and they can behave as stress concentrators leading to premature failure. In this case, it is considered unsafe to use only the yield strength of the manufactured composites in the design of concerning components. Because, fracture can take place in the presence of cracks at smaller loads. Therefore, using fracture mechanics properties is considered the best option to come through such problems [6]. Hence, examination of fracture behavior of these composites is required to assess the reliability of these structures. Furthermore, to the best of authors’ knowledge, fracture toughness of the recycled constituent composites has not been investigated in detail in the literature.

In the frame of this research, after manufacturing these novel composites, three-point bending (3PB) tests were used to determine fundamental mechanical characteristics and the results were verified with Halpin–Tsai analytical modelling. In addition, composite fracture toughness was examined using notched specimens. Then, a practical numerical study was carried out to observe the evolution of the energy release rate along the crack front. In the end, SEM fractography was conducted to examine the toughening mechanisms.

## 2. Experimental Procedure 

### 2.1. Materials

In this study, as raw materials, alumina fibers, recycled EPDM (ethylene propylene diene monomer) rubbers and epoxy matrix were used. Alumina fibers used in this research were procured from Goodfellow SARL (Lille, France) and average dimensions are given as 60–90 μm in length and 10–20 μm in diameter. In terms of the material properties, AF’s tensile modulus is given as 380 GPa and the density of AF is specified as 3.9 g/cm^3^. As the matrix, Araldite DBF epoxy resin was obtained from Huntsman™ (Basel, Switzerland) together with its hardener Aradur HY 956 EN. 20 pbw Aradur HY 956 EN liquid hardener was used for 100 pbw Araldite DBF liquid resin as recommended in supplier’s data sheet. Araldite DBF has a tensile modulus equals to 1465 MPa with a density of 1.1 g/cm^3^. In addition, epoxy content of Araldite DBF is given as 4.20–4.35 Eq/kg. Also, the dynamic viscosity of the liquid epoxy resin modified by the addition of Aradur HY 956 EN is given as 1800 MPa·s at 25 °C [7]. In terms of mechanical properties, Araldite DBF has a tensile modulus equals to 1465 MPa with a density 1.1 g/cm^3^. Lastly, EPDM recycled rubbers which are vulcanized by sulfur were supplied by a sportive equipment manufacturer in Sofia, Bulgaria as fresh scrap. It means that they are collected directly from the production line as waste parts and no contaminants were found in rubber such as metallic particles which could oxidize and overheat the rubber or they may degrade the adhesion of rubber with the matrix. The average diameter of the rubbers is given 10.44 μm, Young’s modulus is indicated as 6 MPa and their density is stated as 1.4 g/cm^3^.

### 2.2. Materials Processing and Experimental Characterization Procedures

**Figure 1:**
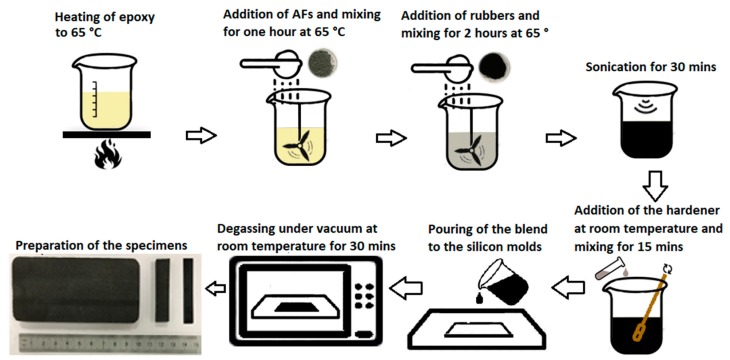
Manufacturing flow chart for the recycled EPDM rubbers modified epoxy-based composites [5].

Manufacturing procedure of the composites is illustrated in Figure 1 and more details can be found in our previous paper [5]. In Figure 1, sonication of AF has a crucial effect on the distribution of the AF. Moreover, degassing of the molded final composite is required to eliminate the formation of air bubbles during the polymerization of epoxy.

In composite manufacturing, the wettability of the used fibers has an influence on the final material properties of the composite. Throughout the literary review of this study, it is seen that AF have a good wettability with epoxy matrix. However, interface characteristics were observed by a cleanly broken polished cross-section of the composites after the manufacturing procedures [8]. By the observation of a satisfying bonding of the fibers to the matrix, no further surface treatment was applied in order not to reduce the cost-efficiency of the composites. Moreover, in the previous studies of the authors [9] vinyltriethoxysilane was used as a surfactant to provide a better adhesion between epoxy–recycled SBR rubber blend. Because, the positive outcomes of silane agents were observed on the composites based on recycled SBR rubbers and epoxy resin. However, in the preliminary trials of this study it is not noticed a remarkable difference on the mechanical properties by using silane agents. Therefore, silane coupling agents were not used in this study due to the cost related issues.

In this research, the content of recycled rubbers and alumina fibers added to epoxy were varied to study their effects on mechanical properties. The compositions of the composites (referred as LRAL composites hereafter) used in the study are given in Table 1.

After manufacturing of the composites, densities were measured with a pycnometer and then quasi-static three points bending tests (3PB – Instron 5569, Norwood, MA, USA) were performed by respecting ASTM D790 standard. Deflection of the specimen was measured by the crosshead position. In addition, fracture toughness parameters such as critical stress intensity factor (*K_Ic_*) and critical strain energy release rate (*G_Ic_*) were investigated with single edge notched beam (SENB) specimens according to ASTM D5045 standard. At least five specimens for each composition were used. Following to mechanical testing, SEM fractography (Scope/JSM-6010LA Jeol®, Tokyo, Japan) was done to identify the toughening and damage mechanisms. 

## 3. Results and Discussions

### 3.1. Experimental Characterization of the Manufactured Composites 

The measured densities of the LRAL composites are given in Table 2. Densities give a direct information about the lightweight features of the composites.

As expected, density of the composites decreased with the increasing rubber content, whereas the density increased by the ascending AF content. After investigating the density, 3PB tests were used to investigate the fundamental mechanical characteristics of the composites.

**Figure 2:**
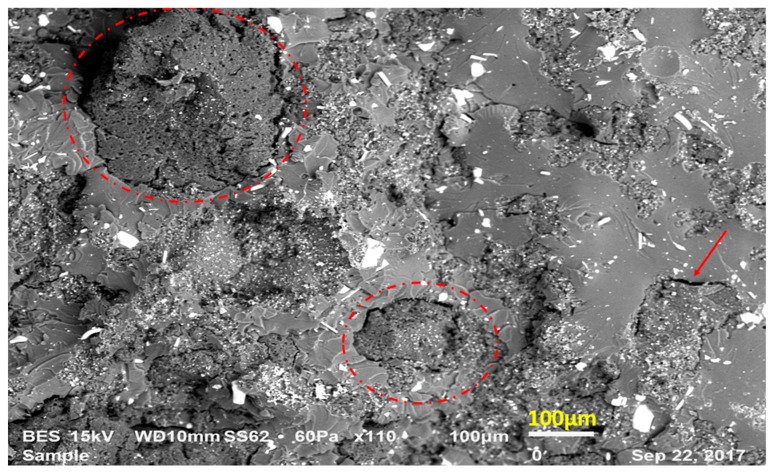
Microscopical observation of the gaps in the epoxy–recycled rubber interfaces.

Averages of the results from the three-point bending test and their standard deviations are given in Table 3. First of all, from Table 3 it can be inferred that AF had no substantial impact on composite strength. Second, increasing rubber content resulted in a drop in both the strain at break and strength of the composites compared to the neat epoxy. This tendency might be correlated with poor interfacial adhesion of the recycled EPDM rubber and epoxy blends. Because of the previous vulcanization process implemented by the recycled EPDM rubber supplier, recycled EPDM rubber particles have no free links on their surfaces. Accordingly, having a chemical bond between recycled rubber and epoxy is difficult. Moreover, voids can be observed at the interfaces due to the insufficient compatibility between recycled EPDM rubber and epoxy, and this causes reduced transfer of stress from the matrix to the rubber particles bringing decreased overall rigidity of the compounds. Besides, in the course of the solidification, different contractions of epoxy and rubber can lead to void formation in the epoxy–rubber interface. As a result, when these newly manufactured composites are exposed to loads, the mentioned voids generate the feeble points of the composite and crack propagation is promoted along these weak boundaries. This causes premature failure. In Figure 2, the fracture surface of a 20 wt. % rubber containing composition is given in order to justify these arguments. In this figure, smooth parts show epoxy whereas rough parts indicate recycled rubbers. 

**Figure 3:**
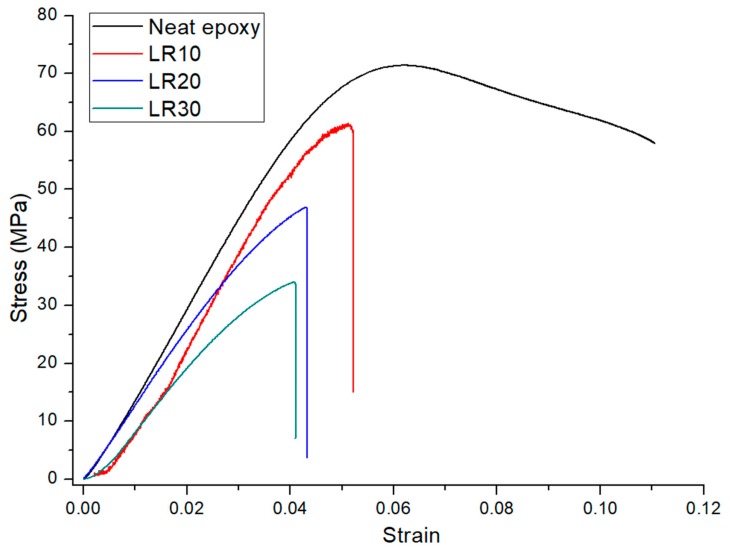
Stress–strain curve of recycled EPDM rubbers modified epoxy-based composites.

Because of the reasons cited above, the number of the discontinuities increases by the ascending rubber content which leads to the drop of strength and strain at break of the composites in consideration. Figure 3 is given to illustrate the effect of the increasing rubber content on the stress–strain curve of the recycled EPDM rubber containing binary composites (LR groups). 

In Figure 3, rubber modified composites are failing abruptly compared to neat epoxy which is an indicator of brittle behavior of the recycled rubber modified composites. Also, increasing content of the recycled EPDM rubbers gradually decreases the strain at break. On the other hand, neat epoxy remains in plastic region for a considerable time before the fracture. Therefore, from Figure 3 it is inferred that recycled rubbers reduce the deformation capability of these composites. To compensate these adverse impacts of the recycled rubber, AF were added to the composite system. The elasticity modulus of the composites is enhanced under favor of the rigid nature of the AF. However, AF could not effectively compensate for possible adverse effects of recycled rubber in strain at break and maximum strength [10,11]. As an intermediate result, for the applications which require high mechanical resistance, the use of 30 wt. % EPDM rubber containing groups (LR3AL compositions) can be avoided.

Moreover, to verify the significance of the AF and recycled EPDM rubber incorporation on the mechanical properties, two-way ANOVA statistical analyses were implemented on the results reported in Table 3 by using Minitab 17 software. During two-way ANOVA, effect of recycled EPDM rubbers (R wt. %), effect of AF (AF wt. %) and effect of the interaction between recycled EPDM rubbers and AF (R × AF wt. %) on ultimate flexural stress, flexural modulus and strain at break were examined independently. The results of two-way ANOVA are given in Table 4. In Table 4, DF indicates degrees of freedom, SS shows sum of squares, MS indicates mean square. Moreover, F and P values are essential to decide whether the significance of the recycled EPDM rubber and AF content have an effect on the mechanical properties, because F and P values are used to establish null hypotheses. After executing two-way ANOVA on Minitab, degrees of freedom in the denominator (degrees of freedom for error) was found. This value was used to determine the critical F-value by using an F-table [12]. If the F-value shown in Table 4 is greater than the F-value determined by using the F-table, null hypotheses are rejected. Second, the P-value is also used to decide the validity of the null hypotheses. If the P-value shown in Table 4 for a selected factor is smaller than 0.001, it can be said that the factor has a significant effect on the examined mechanical property. For instance, in this study when the effect of rubber content on the ultimate flexural stress is investigated, after executing two-way ANOVA, degrees of freedom in the numerator (R wt. %) was found “2” as shown in Table 4 whereas degrees of freedom in the denominator was found as 39. By using F-Table, critical F value was determined as 8.27. The F-value seen in Table 4 (=322.26) is greater than the critical F value (=8.27). In this case, null hypotheses can be rejected. 

After implementing the same procedure on AF content as well as the interaction between AF and recycled EPDM rubber content in the composites, significance of the constituent content on the mechanical properties was determined. Data given in Table 4 indicated that recycled EPDM rubber content, AF content and the interaction between recycled EPDM rubber and AF have significant effects on ultimate flexural stress, flexural modulus and strain at break.

After examining the significance of the constituent contents on the mechanical properties, analytical modelling was used to compare the experimental results. Here, Halpin–Tsai (H–T) model was used for the fiber form reinforcements. This model is convenient to use and mostly good at estimating the experimental results in case of homogeneous distribution of the fillers. Halpin–Tsai has a lot of examples in the literature on hard filler reinforced composites. However, in the case of using two scales of fillers such as AF, the classical H–T model must be modified to consider the effects of fibers more precisely. As two different fillers were used, it was necessary to use the H–T equations twice [13,14]. For AF-reinforced composites, the modified H–T equations are given below:

(1) $$\frac{E_{m1}}{E_{matrix}}=\frac{3}{8}\;\left(\frac{1+\xi_{AF}\eta_{AF-L}\varphi_{AF}}{1-\eta_{AF-L}\varphi_{AF}}\right)+\frac{5}{8}\;\left(\frac{1+2\eta_{AF-T}\varphi_{AF}}{1-\eta_{AF-T}\varphi_{AF}}\right),$$

(2) $$\eta_{AF-L}=\frac{E_{AF}/E_{matrix}-1}{E_{AF}/E_{matrix}+\xi_{AF}},$$

(3) $$\eta_{AF-T}=\frac{E_{AF}/E_{matrix}-1}{E_{AF}/E_{matrix}+2},$$

(4) $$\xi_{AF}=2\;\frac{l_{AF}}{d_{AF}}.$$

In these equations, *E_m_*, *E_AF_*, *E_m1_* are the elasticity modulus of the matrix (=1.465 GPa), alumina fibers (=380 GPa) and AF—epoxy blend respectively. *φ_AF_* is the volume fraction of AF and *l_AF_* and *d_AF_* are the average length (=76 μm) and average diameter (=15 μm) of AF, respectively. In these equations, stress portioning factor *η* was examined as two different terms. *η_AF-L_* indicates the elongated effect of AF whereas *η_AF-T_* covers the transversal effect of the AF. Lastly, *ξ* is denoted as fibers’ shape factor. On the other hand, to estimate the Young’s modulus of epoxy–recycled rubber blend classical H–T equations were used as below:

(5) $$\frac{E_{m2}}{E_{matrix}}=\frac{1+\xi \eta \varphi_{R}}{1-\xi \eta \varphi_{R}},\;\eta =\frac{\frac{E_{R}}{E_{matrix}}-1}{\frac{E_{R}}{E_{matrix}}+\xi }.$$

Here, *E_m2_*, *E_R_* and *E_matrix_* are elasticity modulus of the epoxy-rubber blend, recycled rubbers and the epoxy matrix, respectively and *E_R_* was taken as 6 MPa. *φ_R_* is the volume fraction of the rubbers ξ is again the shape factor of the rubbers is assumed as 2 for the spherical particles. Then, the Young’s modulus of the composite is obtained by the combination of Equations (1) and (5) as given below:

(6) $$E_{composite}=E_{matrix}\times \left[\frac{3}{8}\;\left(\frac{1+\xi_{AF}\eta_{AF-L}\varphi_{AF}}{1-\eta_{AF-L}\varphi_{AF}}\right)+\frac{5}{8}\;\left(\frac{1+2\eta_{AF-T}\varphi_{AF}}{1-\eta_{AF-T}\varphi_{AF}}\right)\right]\times \left[\frac{1+\xi \eta \varphi_{R}}{1-\xi \eta \varphi_{R}}\right].$$

**Figure 4:**
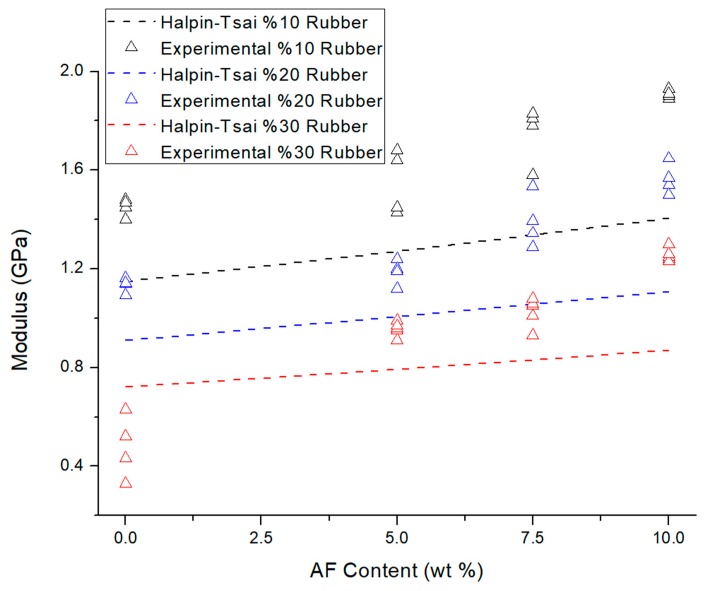
Experimental results and Halpin–Tsai model comparison on elasticity modulus of LRAL composites by the increasing content of AF.

As shown in Figure 4, AF are considerably improving the Young’s modulus also numerically. This improvement is arising from the high modulus of fibers. According to Figure 4, H–T model underestimates the modulus of the manufactured composites. In other words, generally experimental values are seen above the theoretical line and this can be related to the synergetic effects of AF together with recycled rubbers.

**Figure 5:**
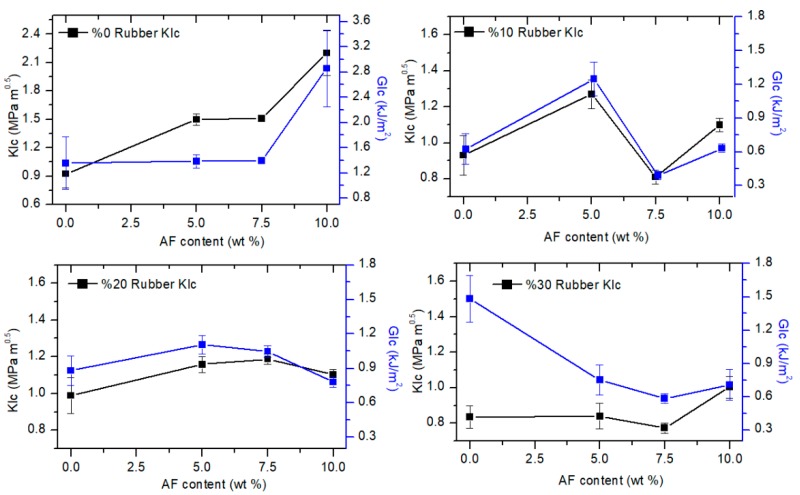
Fracture toughness test results of the manufactured composites.

After the analysis of principal mechanical characteristics, fracture toughness of the composites was also studied. Fracture toughness is a numerical designation of the resistance of material to crack propagation under load. In Figure 5, *K_Ic_* and *G_Ic_* of the manufactured composites are given. 

From Figure 5, a lot of fluctuations on the fracture toughness attract attention. At first, alumina addition to rubber and epoxy combination leads to an increase in toughness. In 10 wt. % and 20 wt. % rubber contents, this positive effect is quite apparent. However, for 30 wt. % of rubber consisting ternary composites, at least 10 wt. % of alumina is required to compensate the detrimental effect of rubbers on fracture toughness. Also, in certain compositions, AF reinforcement does not improve the composites’ fracture toughness. This may stem from the internal defects on the specimens because test specimens are extracted from the same composite block, and internal defects can be valid for each of them. Therefore, in the presence of the defects, composite fails from the weakest part of the specimen which significantly decreases the fracture toughness of the composite [15]. On the side of fracture energy, even if it is difficult to establish a straightforward trend, incorporation of AF to the epoxy/recycled rubber blend generally enhances the fracture energy except for 30 wt. % of rubber containing compositions. This situation can arise from low maximum strength values. As formerly mentioned, the area under the stress–strain curve is a direct function of the energy absorbing capacity of the material. In these composites, with the increasing content of rubbers, stress values are declining considerably and the added AF cannot compensate this drop. In addition, AF do not ensure a significant improvement in strain at break. Hence, the area under the stress-strain curve in other words, fracture energy remains smaller in LR30AL group composites compared to 10 and 20 wt. % rubber modified ones.

Lastly, in the right bottom of Figure 5, *K_Ic_* and *G_Ic_* of LR3AL5 are seen inversely proportional to each other. This is observed due to the high difference in the elasticity modulus between LR30–LR3AL5. Because, elasticity modulus is the denominator in the *G_Ic_* equation and for this reason, the division of the numerator by a grand denominator leads to this illusion.

**Figure 6:**
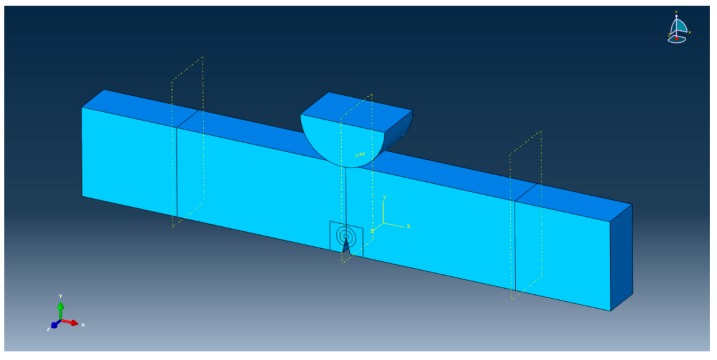
Assembly of the simulated test specimen.

After the experimental examination of the fracture toughness, a practical numerical approach was carried out to observe the evolution of the energy release rate (*J*-integral) along the specimen thickness. In the literature, various researchers used *J*-integral as a substitute for *G_Ic_* and this is also maintained in this study [16,17]. The model employed here was developed and described in detail. Figure 6 shows the solid model of the SENB specimen used during FEM analysis. In this model the loading support (upper semi-cylinder) was designed as a part of the assembly to eliminate stress concentration above the crack tip. The vicinity of the crack tip was examined in three circular partitions, to refine the mesh around the crack. 

**Figure 7:**
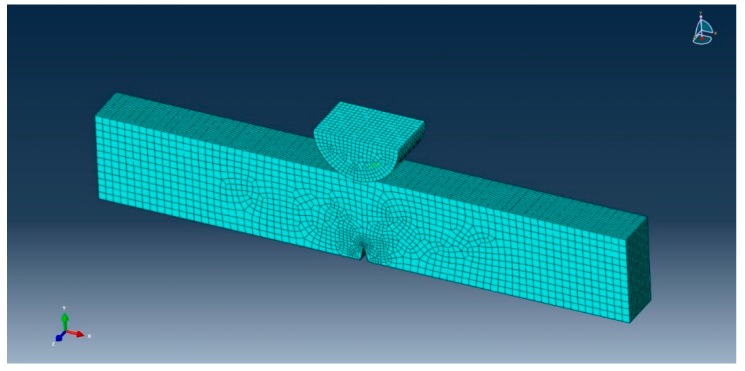
Meshed specimen.

In the same way, Figure 7 indicates the mesh of the specimen and the refined center where the crack propagates. The refined mesh was about 0.09 × 0.11 × 0.16 mm^3^. We used 86200 linear hexahedral elements of type C3D8R in the models. A reference point was created on the loading support in order to impose the associated displacement at the fracture.

**Figure 8:**
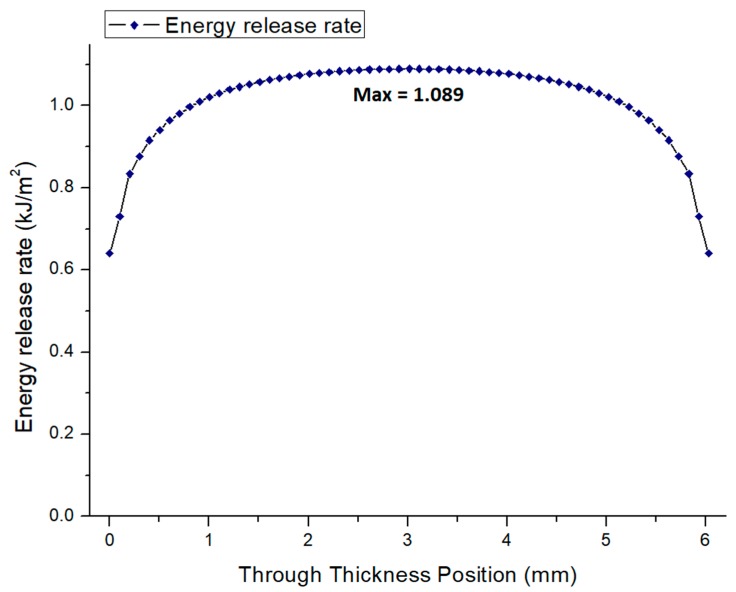
Evolution of the energy release rate in mode I for the SENB specimen in the thickness.

By using the basics of linear elastic fracture mechanics in this model, at first, an experimentally obtained displacement value causing the fracture of the specimen was applied to the material as a boundary condition. This loading leads to mode I crack opening, and elasticity modulus was set as obtained from the experiments. After the execution of the model in a single load step with a time period of 1, total force occurring in the load pin was determined. After the comparison of this reaction force with the experimental value, the proportion of them was used to adjust more precisely the selected elasticity modulus. By doing so, an estimation for the local elasticity modulus was done. After the modification of the elasticity modulus on Abaqus, the model was simulated again with an initial time increment of 10^−3^. This iterative modification of elasticity modulus was maintained until a consistent maximum force with respect to experimental results. After converging to the maximum force on Abaqus as in the experiments, energy release rate (*J*-integral) of the composites was determined along the crack front or in other word in specimen thickness. In fact, a major part of the experimental approaches defines the energy release rate as a function of *K_Ic_* with a single value depending on the plane stress or plane strain condition. This means that tri-axial effects or 3-D constraint effects are generally ignored. In this case, failure prediction in both cleavage and ductile fracture may be error-prone. However, in case of a high reliability, uncertainties limit the assessment of the structures. Therefore, complete behavior of energy release rate along the crack front should be obtained through 3D analysis. In this regard, one of the compositions (here selected as LR1AL5) used in fracture tests, numerically tested according to the above-mentioned model. Experimental data for the numerically tested specimen is given in Table 5. After executing this model, the focus was given to the evolution of the energy release rate along the crack front and the results are presented in Figure 8.

Figure 8 represents the evolution of the energy release rate along the crack front of the specimen. It clearly shows that the energy release rate for the SENB specimen is not constant along the thickness. Because, in the middle of the specimen thickness or in other words, inside the specimen, the presence of $\sigma_{zz}$ leads to a high level of triaxiality. Therefore, plane strain stress state is observed in the central region of the specimen. However, in the free surfaces of the specimen, the stress triaxiality is removed and pure plane stress state is experienced. Thus, in the central part of the specimen, plane strain conditions due to the triaxial stress state cause higher stresses in the crack zone. This higher stress state leads to crack formation and then propagation leading to the failure. In addition, coalescence of the micro voids under triaxiality may contribute to the failure. Lastly, the presence of different levels of out of plane constraint (out of plane tensile load) may lead to the variation on the energy release rate curve [18,19].

In terms of the values obtained from the model, according to a conservative approach, the maximum value of the curve can be used as a measure of the material’s fracture toughness. In the tests, strain energy was obtained as 1.14 kJ/m^2^ whereas in the model it is observed at a maximum value of 1.089 kJ/m^2^. The error between these values is calculated as 4.5%. In the model, the presented values in Figure 8 was taken from the surface but due to the mesh sensitivity issues numerical solutions may show differences. In summary, here in this study, the focus was given to the evolution of the energy release rate by performing a practical finite element modelling. In this modelling, no plasticity and no shear effects were considered. Also, shape effects of the constituents were neglected. Therefore, there is an evident need for further studies to simulate more closely the real fracture behavior of these composites.

### 3.2. Toughening Mechanisms Identification by Means of SEM

After mechanical tests, to identify various toughening mechanisms, SEM observations were performed on the fracture surface of the failed specimens during 3PB tests.

**Figure 9:**
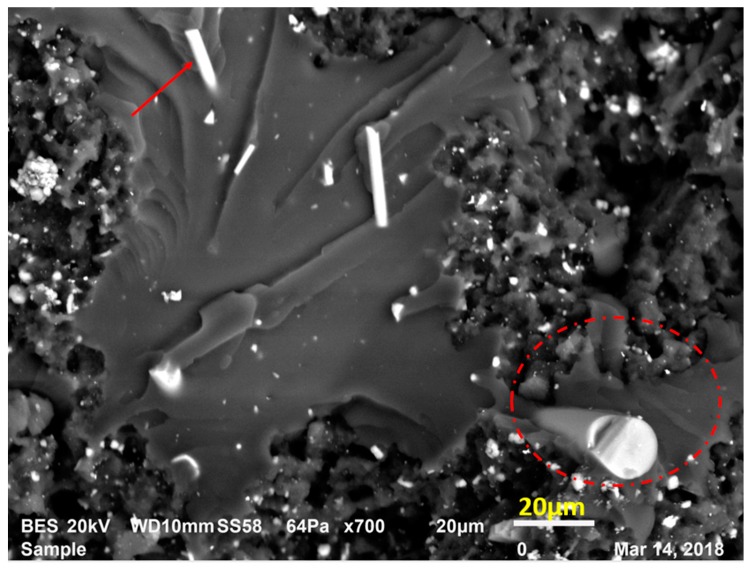
Fiber bridging mechanism in LRAL group composites.

First toughening mechanism was observed as fiber bridging. In Figure 9, red arrows and circles are showing some fibers with different dimensions on the fracture surface. The fibers aligning near normal to the fracture surface plane or the crack opening play most probably a bridge role. This implies that at each bridging site the fiber produces a discrete traction which reduces the generated stress at the crack tip by the virtue of increasing fracture toughness. In this manner, crack opening can be obstructed by fiber bridging in the zone of crack propagation. For example, to propagate the crack, more strain energy is needed to be applied and subsequently dissipated to surmount the fiber bridging constraint. Thus, fracture toughness is improved [20,21,22].

**Figure 10:**
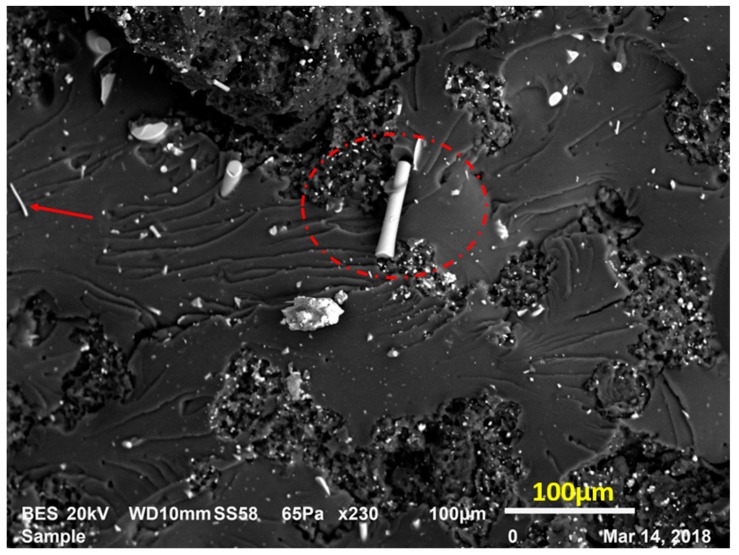
Fiber pull-out in LRAL group composites.

Moreover, strain softening is seen in epoxy after yielding, which is widely seen in glassy polymers, accordingly the yielded epoxy matrix shall be relatively compliant and its plastic deformation occurs more smoothly. However, the “rigid” AF will obstruct any important plastic dilatation in epoxy, unless these AF debond from the epoxy matrix. Therefore, some of the stored strain energy is dissipated by the interfacial debonding of AF. In Figure 10, some of the attached fibers on the fracture surface (displayed with a red arrow) show a significant length out of the fracture zone, and the fiber was oriented at an angle to the crack propagation direction. This implies that this fiber was pulled-out in the course of crack propagation. This is accepted as another damage mechanism known as fiber pull-out [23,24]. 

**Figure 11:**
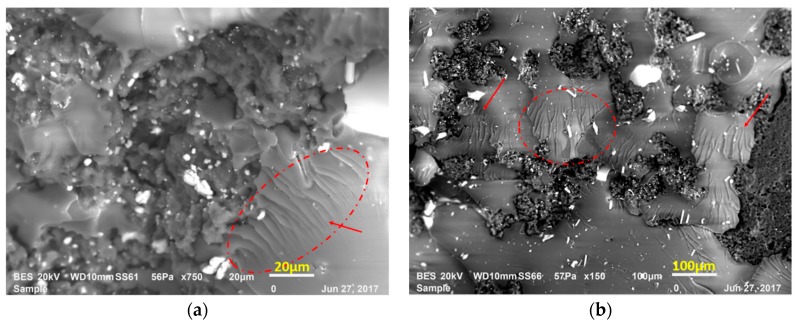
Shear yielding in LRAL composites. (**a**) LR2AL5 group, (**b**) LR2AL7.5 group.

Fiber pull-out is generally accompanied by shear yielding and shear band formation of the epoxy matrix as shown in Figure 11a,b. In Figure 11a,b shear yielding indicated with shear bands marked with the red arrows. Shear yielding can increase the strength of the polymers significantly in the absence of brittle fracture. Even though the final material reveals a brittle fracture, polymer matrix provides a localized plastic and viscoelastic energy dissipating process around the crack tip during the crack propagation. In consequence, shear yielding contributes to the energy absorption in the polymer matrix and increases the fracture toughness of the composite [25,26,27].

Fracture surface observation on the ternary composites showed different toughening mechanisms provided by AF and rubbers. AF bridging was seen as the main mechanisms that improve the composites’ fracture toughness. In addition, shear yielding is promoted by the presence of the recycled rubbers and AF. In conclusion, a good combination of different toughening mechanisms improved the fracture toughness of the manufactured composites.

## 4. Conclusions

A comprehensive work was carried out for a new design of recycled rubber modified epoxy-based composites. These cost-effective composites were reinforced with fine alumina fiber. The main idea was to evaluate the fracture behavior and damage analyses. Essentially, 3PB tests have shown that recycled EPDM rubbers have a tendency to degrade the mechanical resistance and strain values at failure of these composites. However, the reinforcement of fine alumina fiber in general way improves the value of the elasticity moduli. SEM failure analyses have exposed well a fiber bridging, pull-out of fibers during the deformation as toughening mechanisms in these composites. 

Additionally, experimental results were compared with a modified “Halpin–Tsai” model and the results were related to the synergetic effects of alumina fiber together with recycled rubbers. Evidently, the fracture toughness values have shown some variabilities due to local clusters in the microstructure at high level of the reinforcements under laboratory conditions. 

Numerical analysis of the energy release rate has revealed a remarkable stress state dependency along the crack front. Future work will address this area of research.

These composites will be used in the manufacturing of suspension systems and fuel tank mountings, basically in the automotive engineering applications.

## Figures and Tables

**Table 1:** Composition of AF reinforced recycled EPDM rubber blended epoxy-based composites.

LRAL Composites	Alumina Fiber Content (wt. %)			
Rubber content (wt. %)	0%	5%	7.50%	10%
10%	LR10	LR1AL5	LR1AL7.5	LR1AL10
20%	LR20	LR2AL5	LR2AL7.5	LR2AL10
30%	LR30	LR3AL5	LR3AL7.5	LR3AL10

**Table 2:** Density of the manufactured composites.

Composition Name	Density (g/cm^3^)	Composition Name	Density (g/cm^3^)	Composition Name	Density (g/cm^3^)
LR10	1.120	LR20	1.115	LR30	1.035
LR1AL5	1.140	LR2AL5	1.129	LR3AL5	1.060
LR1AL7.5	1.151	LR2AL7.5	1.135	LR3AL7.5	1.089
LR1AL10	1.176	LR2AL10	1.141	LR3AL10	1.103

**Table 3:** Mechanical properties of the tested compositions.

Composition Name	Ultimate Flexural Stress (MPa)	Flexural Modulus (MPa)	Strain at Break
Neat epoxy	78.96 ± 1.22	1465.83 ± 145.05	0.13 ± 0.017
LR10	61.58 ± 1.64	1454.71 ± 16.28	0.049 ± 0.002
LR1AL5	55.63 ± 2.05	1552.61 ± 67.64	0.038 ± 0.001
LR1AL7.5	50.73 ± 0.95	1754.25 ± 57.10	0.030 ± 0.001
LR1AL10	49.15 ± 0.35	1911.75 ± 12.02	0.027 ± 0.001
LR20	48.33 ± 1.02	1149.64 ± 20.74	0.045 ± 0.001
LR2AL5	40.17 ± 0.93	1220.77 ± 38.43	0.035 ± 0.001
LR2AL7.5	40.06 ± 1.20	1349.33 ± 42.30	0.037 ± 0.006
LR2AL10	40.97 ± 0.91	1564.50 ± 83.23	0.040 ± 0.001
LR30	34.27 ± 3.77	478.25 ± 64.13	0.037 ± 0.001
LR3AL5	30.61 ± 0.99	962.94 ± 14.23	0.033 ± 0.001
LR3AL7.5	29.59 ± 1.30	1029.57 ± 30.09	0.030 ± 0.001
LR3AL10	39.75 ± 0.60	1259.82 ± 13.64	0.033 ± 0.001

**Table 4:** Two-way ANOVA results on the mechanical properties of the composites.

Two-Way ANOVA	Ultimate Flexural Stress			Flexural Modulus			Strain at Break		
	R wt. %	AF wt. %	R × AF wt. %	R wt. %	AF wt. %	R × AF wt. %	R wt. %	AF wt. %	R × AF wt. %
DF	2	3	6	2	3	6	2	3	6
SS	3621.2	288.1	517.5	3,996,251	1,761,240	190,714	0.000147	0.001153	0.000351
MS	1810.58	96.04	86.25	1,998,126	587,080	31,786	0.000074	0.000384	0.000059
F-Value	322.26	17.09	15.35	332.41	97.67	5.29	10.65	55.61	8.47
P-Value	0.000	0.000	0.000	0.000	0.000	0.000	0.000	0.000	0.000

**Table 5:** Characteristics of LR1AL5 used in the tests.

**Thickness (mm)**	9.47
**Width (mm)**	6.03
**Length (mm)**	50
**Notch Length (mm)**	4.20
**Force to Failure (N)**	87
**Elasticity Modulus (MPa)**	1552
